# Allergy Is a Global Problem

**DOI:** 10.1097/wox.0b013e3181651c65

**Published:** 2008-01-15

**Authors:** Johannes Ring

**Affiliations:** 1 Editor-in-Chief, Munich, Germany, January 2008

## 

Twenty years ago, and still in some textbooks, allergy was regarded as a "disease of the rich," with a rising prevalence only in industrialized or western countries. This has changed: allergy has become a major health problem in almost all countries of the world, both in terms of individual suffering, medical management, and quality of life, as well as a socio-economic burden. People in Papua New Guinea or Tanzania are experiencing very similar allergic diseases and symptoms as patients from São Paulo, New York, Moscow, or Tokyo. Yet there are considerable differences in management, diagnostic and therapeutic modalities, and especially with regard to eliciting allergens and modifying environmental factors. Globalization is not only an economic phenomenon. In addition, the world has grown together in science and in the medical field.

There is a need for more information, exchange of experience, and for a discussion forum at an international level. Allergy with its interdisciplinary character involves the expertise of various fields both in clinical medicine and in research and experimental science. Allergy manifests itself as various conditions with a colorful spectrum affecting different organs and following quite variable pathomechanisms.

The World Allergy Organization (WAO), as a global federation of 74 national and regional societies for allergology and immunology, is launching the new journal "WAO Journal," addressing this need for information, exchange of experience, and international discussion.

The journal will be practice-oriented and offer the highest level of information in a strictly peer-reviewed system. It offers space for reviews both at the postgraduate education as at the advanced research level, originals, and letters, where exceptional case studies, concise reports on study findings, or expression of opinions and discussion of recent topics of controversy will be published.

The journal has established an excellent group of experts in the editorial board who represent at the same time the various features of clinical and experimental allergology and relevant subdisciplines and the various geographic areas of the world.

The WAO Journal will reach over 30,000 allergists all over the world directly online; it will be available for free for the first year for all individual members of national allergy societies as well as nonmember subscribers. The new journal is in direct tradition grown on the basis of Allergy and Clinical Immunology International--The Journal of the World Allergy Organization, which has been the official organ of WAO for 19 years and has been discontinued by December 2007. This decision never was caused by dissatisfaction; Allergy and Clinical Immunology International--The Journal of the World Allergy Organization was one of the most successful allergy journals, at one time reaching 17,000 allergists, with editions in several languages. At this place, special thanks should go to the founding editor, Alain De Weck, and to the long-time editor, Allen Kaplan, who have done an excellent job together with Hogrefe Huber publishers, Dr Christiane Hogrefe, Dr Rob Dimbleby, and Christina Sarembe. The decision for a new journal reflects a new philosophy of publication, concentrating on the electronic way, with many advantages starting from the speed with which articles can be accepted, reviewed, and published, decreased costs, the opportunity for digital content (including videos and animation), easy searching of contents, and independence from postal services, just to name a few.

Coeditor in Chief Lanny Rosenwasser and myself are eager to push "WAO Journal" to become one of the premium international journals in allergy in a truly global endeavor!

Johannes Ring

Editor-in-Chief

Munich, Germany, January 2008

